# Femoral posture during embryonic and early fetal development: An analysis using landmarks on the cartilaginous skeletons of *ex vivo* human specimens

**DOI:** 10.1371/journal.pone.0285190

**Published:** 2023-05-02

**Authors:** Tetsuya Takakuwa, Marie Ange Saizonou, Sena Fujii, Yousuke Kumano, Aoi Ishikawa, Tomoki Aoyama, Hirohiko Imai, Shigehito Yamada, Toru Kanahashi

**Affiliations:** 1 Human Health Science, Graduate School of Medicine, Kyoto University, Kyoto, Japan; 2 Department of Systems Science, Graduate School of Informatics, Kyoto University, Kyoto, Japan; 3 Congenital Anomaly Research Center, Graduate School of Medicine, Kyoto University, Kyoto, Japan; Brunel University London, UNITED KINGDOM

## Abstract

The pre-axial border medially moves between the fetal and early postnatal periods, and the foot sole can be placed on the ground. Nonetheless, the precise timeline when this posture is achieved remains poorly understood. The hip joint is the most freely movable joint in the lower limbs and largely determines the lower-limb posture. The present study aimed to establish a timeline of lower-limb development using a precise measurement of femoral posture. Magnetic resonance images of 157 human embryonic samples (Carnegie stages [CS] 19–23) and 18 fetal samples (crown rump length: 37.2–225 mm) from the Kyoto Collection were obtained. Three-dimensional coordinates of eight selected landmarks in the lower limbs and pelvis were used to calculate the femoral posture. Hip flexion was approximately 14° at CS19 and gradually increased to approximately 65° at CS23; the flexion angle ranged from 90° to 120° during the fetal period. Hip joint abduction was approximately 78° at CS19 and gradually decreased to approximately 27° at CS23; the average angle was approximately 13° during the fetal period. Lateral rotation was greater than 90° at CS19 and CS21 and decreased to approximately 65° at CS23; the average angle was approximately 43° during the fetal period. During the embryonic period, three posture parameters (namely, flexion, abduction, and lateral rotation of the hip) were linearly correlated with each other, suggesting that the femoral posture at each stage was three-dimensionally constant and exhibited gradual and smooth change according to growth. During the fetal period, these parameters varied among individuals, with no obvious trend. Our study has merits in that lengths and angles were measured on anatomical landmarks of the skeletal system. Our obtained data may contribute to understanding development from anatomical aspects and provide valuable insights for clinical application.

## 1. Introduction

The Carnegie staging system categorizes the first eight weeks after fertilization into 23 stages and is widely used [1, 2]. Staging is based on external appearance and internal findings. Because limb development is a conspicuous and potentially helpful marker for the identification of normal development, the staging system therefore includes limb findings. The upper-limb bud emerging at Carnegie stage (CS) 12 is the first externally detectable finding during human development [1, 2]. The lower-limb bud subsequently appears following the emergence of the upper-limb bud, with a lag of one or two stages (i.e., at CS13 or CS14). Until CS19, the axes of both the upper and lower limbs are more or less parallel. Pre-axial and post-axial borders can be identified, which are cephalic and caudal to the longitudinal axis, respectively. The future thumb and big toe are located on the pre-axial border of the hand and footplates, respectively.

The pre-axial border medially moves between the fetal and early postnatal periods, and the foot sole can be placed on the ground [3]. Several anatomical structures observed in adult lower limbs indicate the medial rotation of the lower limbs during development. For instance, the segmental pattern of lower-limb innervation twists into a spiral [2], and three capsular ligaments (namely, the iliac, pubic, and ischiofemoral ligaments) around the hip joint run spirally [4]. In symmelia, the post-axial side, including the fibula, merges medially. Considering that symmelia becomes visible prior to the end of the embryonic period (i.e., CS23), medial rotation is thought to begin during the embryonic period [3, 5]. Nonetheless, the precise timeline during which this posture is achieved remains poorly understood.

**Fig 1:**
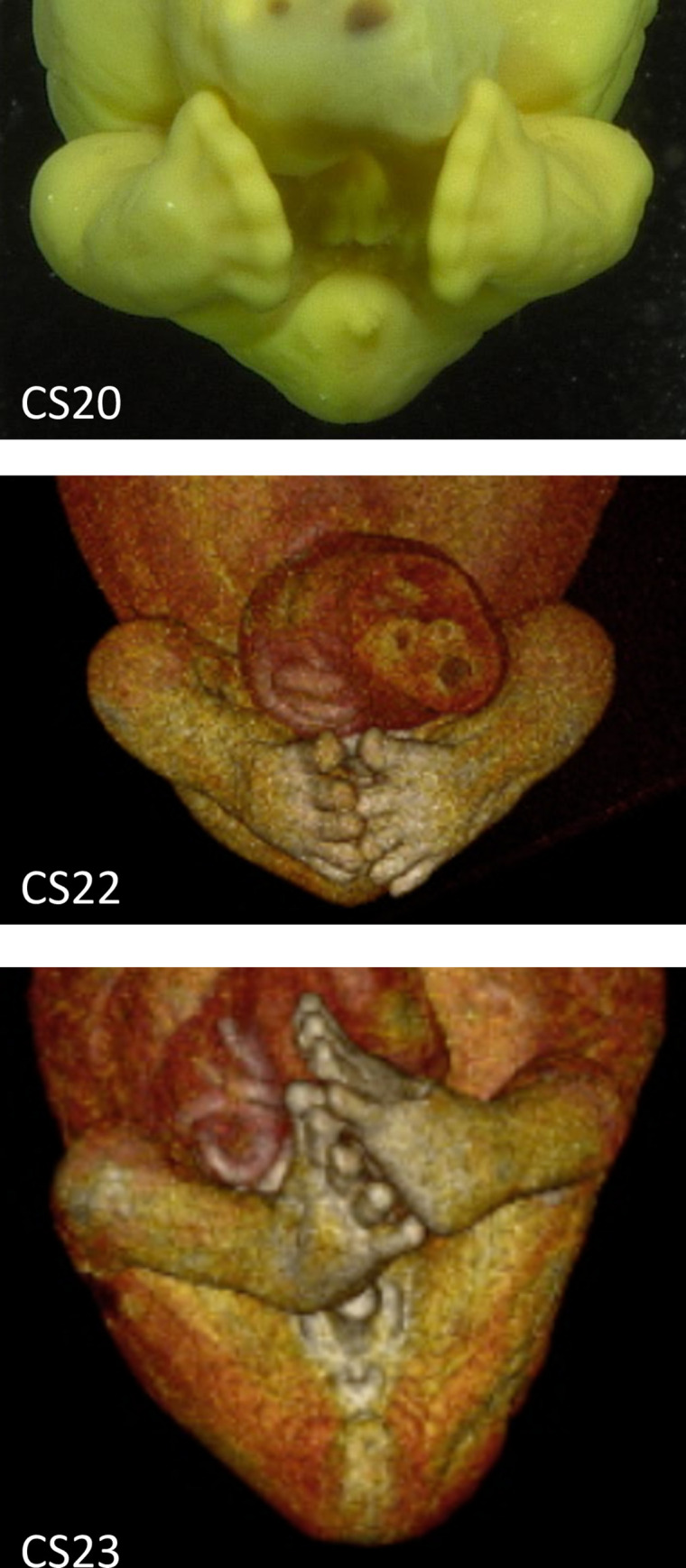
Representative ventral image showing the lower-limb posture during the late embryonic period. Note that the impressive “praying feet” posture (i.e., the hallux was located on the cranial side, and the foot sole oriented medially) was observed at CS23 or earlier stages. The hip joint showed <90° of lateral rotation, whereas the ankle showed adduction. Abbreviation: CS, Carnegie stage.

O’Rahilly and Gardner estimated the lower-limb rotation using toe and foot orientations as reference points; they described that the hallux was located on the cranial side and that the foot sole oriented medially at CS23 [3], which is referred to as the famous “praying feet” posture (Fig 1). Thus, they considered that the pre-axial border remained cranial at the end of the embryonic period (i.e., CS23). During the late embryonic and early fetal periods, the limbs markedly increase in length and exhibit more advanced differentiation in their subdivisions [6, 7]. The hip, knees, ankle joints, and pelvis may contribute to the lower-limb posture. With respect to the ankle joints, physiological clubfoot is recognized during the embryonic and early fetal periods [8, 9]. Physiological clubfoot is a decrease in the foot angle with the frontal side of the leg (plantar flexion), as well as an increase in the foot angle initially, followed by a subsequent decrease in the foot angle with the lateral side of the leg (adduction) (Fig 1). Therefore, the methods of O’Rahilly and Gardner [3] for estimating the timeline of lower-limb rotation seem inappropriate.

There has been remarkable progress in the visualization of the developing fetus owing to recent developments in three-dimensional (3D) sonographic imaging techniques [8, 10, 11]. The use of *in vivo* images (e.g., 3D/4D ultrasound images) may be realistic and ideal because they can enable the visualization of the embryonic and fetal posture in the physiological intrauterine environment, as well as the monitoring of the growth and differentiation of the same individuals. However, during the embryonic period, the cartilaginous skeleton is less echogenic, and accurate identification and quantitative measurement of the posture are challenging to accomplish.

Among anatomical structures, the hip joint is the most freely movable in the lower limbs and largely determines the lower-limb posture. No previous studies provided sufficient knowledge about the femoral posture during the embryonic and early fetal period. Hence, the present study aimed to establish a timeline of lower-limb development using a precise measurement of femoral posture. We successfully achieved this by evaluating the 3D position of the femur relative to the body axis (sacrum) using anatomical landmarks on the lower-limb joints.

## 2. Materials and methods

### 2.1. Human embryonic specimens

The ethics committee of Kyoto University Faculty and Graduate School of Medicine approved this study (E986 and R0316), which used human embryonic and fetal specimens.

The samples included 157 human embryonic specimens between CS19 and CS23 (CS19; N = 25, CS20; N = 38, CS21; N = 36, CS22; N = 32, and CS23; N = 26) and 18 fetal specimens (crown rump length [CRL]: 37–225 mm) from the Kyoto Collection at the Congenital Anomaly Research Center of Kyoto University, Japan [12]. The majority of specimens in the Kyoto Collection are stored for research purposes and provided on request; such specimens are acquired when a pregnancy is terminated for socioeconomic reasons under the Maternity Protection Law of Japan. Samples were collected between 1963 and 1995 in accordance with the relevant regulations during these time periods. Written informed consent from parents was not required at that time; instead, the parents provided verbal informed consent for the deposition of specimens, and consent was documented in medical records. All samples were anonymized and de-identified. Approximately 20% of these fetal specimens are undamaged and well-preserved. Specimens of aborted fetuses were brought to the laboratory, where they were measured, examined, and staged using the criteria proposed by O’Rahilly and Müller in 1987 [1]. Staging of the embryos used was performed by TT (author) and Ms. Chigako Uwabe (acknowledged for technical assistance).

### 2.2. Magnetic resonance image processing and selection of datasets

Magnetic resonance imaging (MRI) was performed using a 2.35-T MRI system [13], 7-T MRI system (BioSpec 70/20 USR; Bruker BioSpin MRI GmbH, Ettlingen, Germany), and a 3-T MRI system (MAGNETOM Prisma; Siemens Healthineers, Erlangen, Germany). The image acquisition method was selected according to the desired specimen resolution and volume. The 2.35-T MRI system was used to acquire 3D images of all embryonic samples between CS19 and CS23 [13, 14]. The 7-T MRI system was used to acquire 3D images of fetal specimens with a CRL of 37.2–103 mm, whereas the 3-T MRI system was utilized to obtain 3D images of fetal samples with a CRL of >116 mm. The conditions for acquisition are described elsewhere [15].

In the present study, 3D images of the whole body were automatically obtained, whereas 3D images of the pelvis and femur were manually reconstructed using AMIRA software version (Visage Imaging GmbH, Berlin, Germany).

### 2.3. Definition of landmarks, 3D axis, and measurements

**Fig 2:**
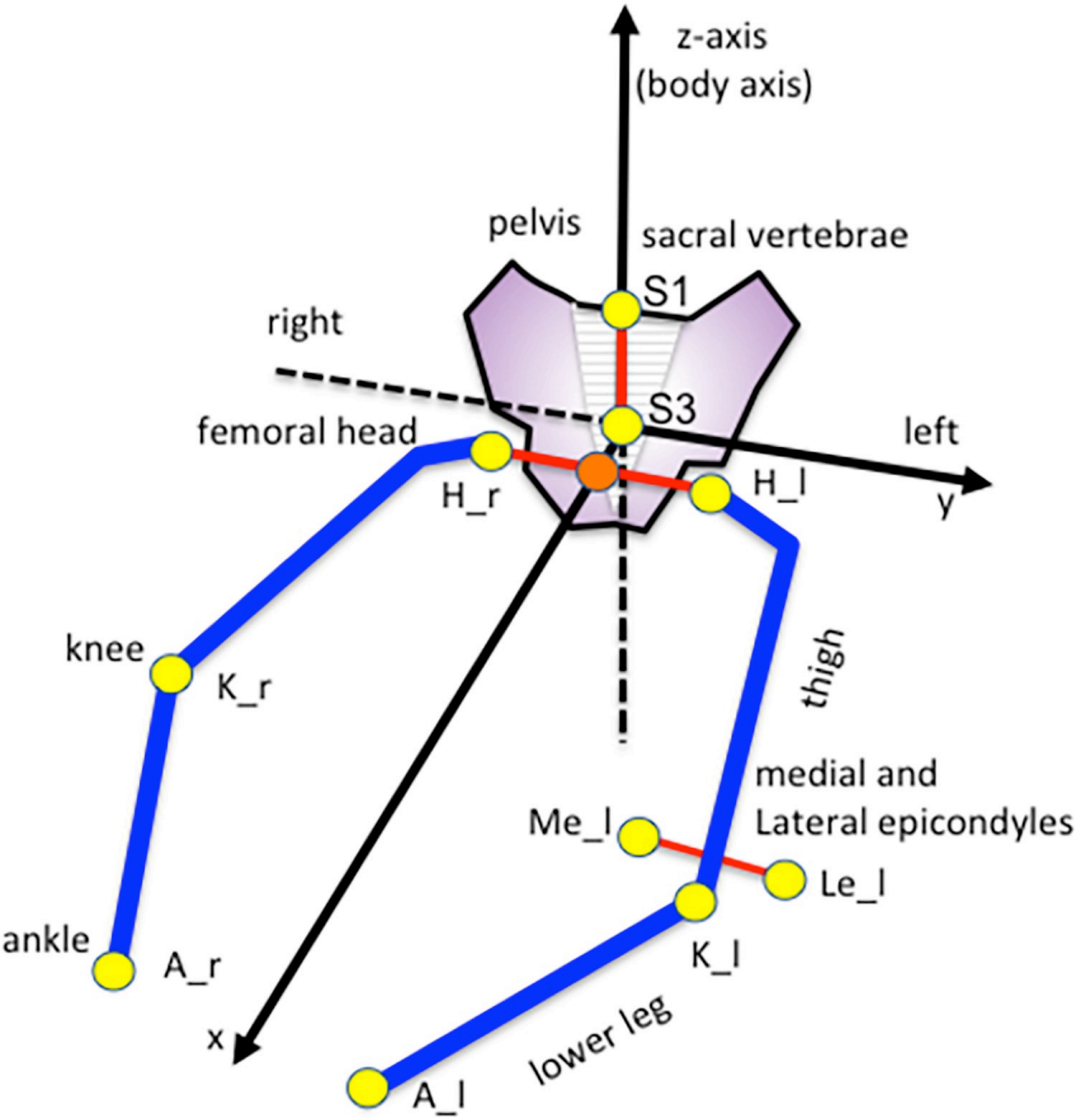
Definitions of the landmarks and coordinate system. For embryonic specimens, the eight selected landmarks were as follows: center of bilateral femoral heads (H_r, H_l); bilateral knee (K_r, K_l) and ankle joints (A_r, A_l); and cranial region of the first and third sacral vertebrae (S1, S3). For fetal specimens, the medial and lateral femoral epicondyles (Me_r, Me_l, Le_r, Le_l) were selected instead of the bilateral ankle joints. S3 was defined as the origin and the z-axis (body axis) as the line through S1 and S3. The z–x plane (median plane) was defined using the midpoint between H_l and H_r (H_m); H_m was located in the median plane. The y-axis was defined as the normal vector of the median plane, and the x-axis was calculated as the outer product of the z- and y-axes.

For embryonic specimens, 3D coordinates were initially assigned to eight selected landmarks by examining the voxel’s position on 2D sequential images and 3D images using Miele-LXIV (Alex Bettarini, https://dicom.3utilities.com/) or AMIRA software (Fig 2). The eight selected landmarks were as follows: center of bilateral femoral heads (H_r, H_l); bilateral knee (K_r, K_l) and ankle joints (A_r, A_l); and cranial region of the first and third sacral vertebrae (S1, S3). For fetal specimens, the medial and lateral femoral epicondyles (Me_r, Me_l, Le_r, Le_l) were selected instead of the bilateral ankle joints.

The original coordinate values were translated into another coordinate system, in which S3 was defined as the origin and the z-axis (body axis) as the line through S1 and S3 (Fig 2). The z–x plane (median plane) was defined using the midpoint between H_l and H_r (H_m); H_m was located in the median plane. The y-axis was defined as the normal vector of the median plane, and the x-axis was calculated as the outer product of the z- and y-axes. Two of the authors (MAS and AI) independently acquired the 3D reconstructions and landmarks. When landmark values were missing or apparently different between two observers, the data were re-acquired. The samples were excluded if the re-acquired data were still discrepant. Consequently, 15 embryonic samples and one fetal sample were excluded from further analysis. The remaining samples had no missing data. Measurements were highly reproducible (intra-class correlation coefficient: 0.90<). The average value for each specimen was used in this study.

The lengths of the bilateral femoral shafts (segments HK_r and HK_l), lower leg (segments KA_r and KA_l), inter-hip joint (segment H_r-H_l), and inter-sacrum S1–S3 were calculated.

The right femoral posture relative to the body axis was defined as the angle between segment HK_r and the z-axis, whereas the left femoral posture was defined as the angle between segment HK_l and the z-axis. These angles were projected onto the median and coronal planes to obtain the flexion and abduction angles. For embryonic samples, the lateral rotation angle of the right femur was calculated using the z-axis, segment HK_r, and vector of plane HKA_r, whereas the lateral rotation angle of the left femur was calculated using the z-axis, segment HK_l, and vector of plane HKA_l. As for the lateral rotation angle for fetal samples, the vector Le-Me was used instead of the vector of plane HKA.

Knee flexion was calculated as the angle between HK_r and KA_r and between HK_l and KA_r. The angle between H_r-S1 and S1-H_l was projected onto the x–y plane (i.e., transverse plane) and was defined as the hip–sacrum angle. The height of the hip joint along the z-axis was calculated by comparing the S1–S3 length. All length and angle values obtained were provided in S1 Datasets.

## 3. Results

### 3.1. Femoral posture

#### 3.1.1. Lateral view (hip and knee flexion)

**Fig 3:**
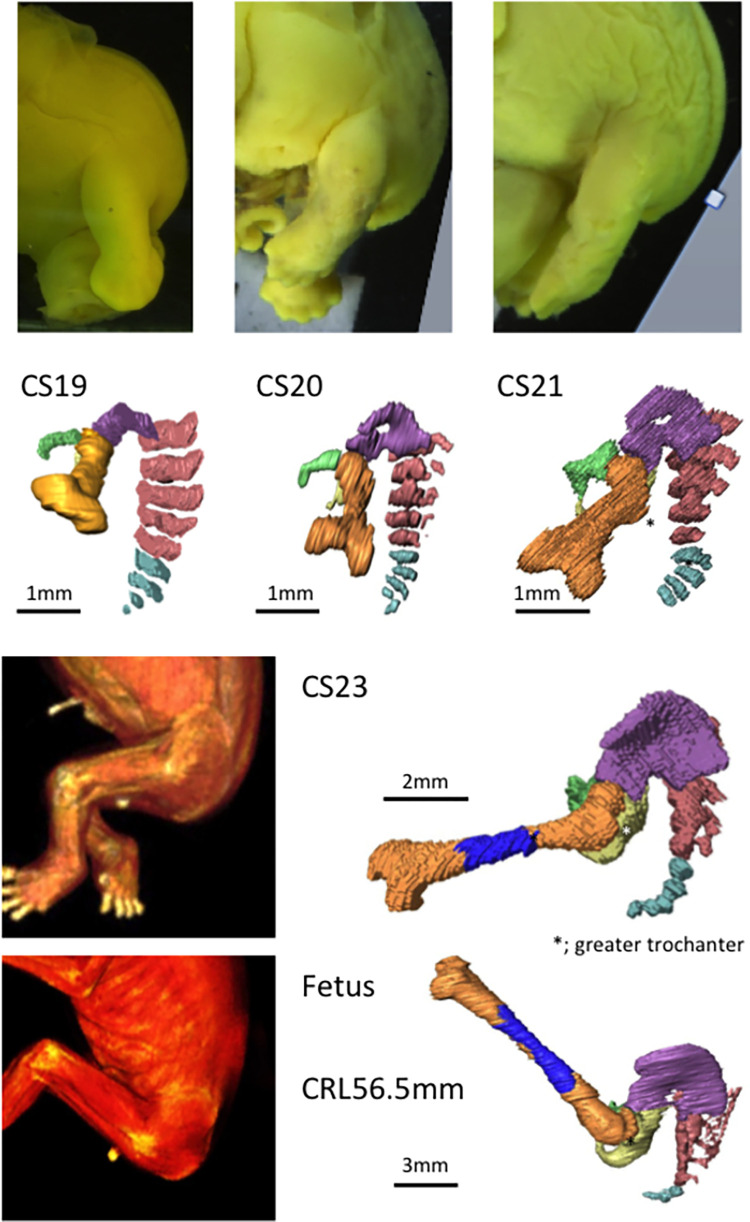
Representative overall pictures and 3D reconstructions of skeletal structures: The hip joint (lateral view) during the embryonic period between CS19 and CS23 and during the early fetal period. Note that the relative position of the hip joint and sacrum in the cranial–caudal direction changed between the embryonic and fetal periods. Legends: blue, femur (ossified); brown, femur (not ossified); green, pubis; light blue, coccyx; pink, sacrum; purple, ilium; yellow, ischium. “*” indicates the greater trochanter.

**Fig 4:**
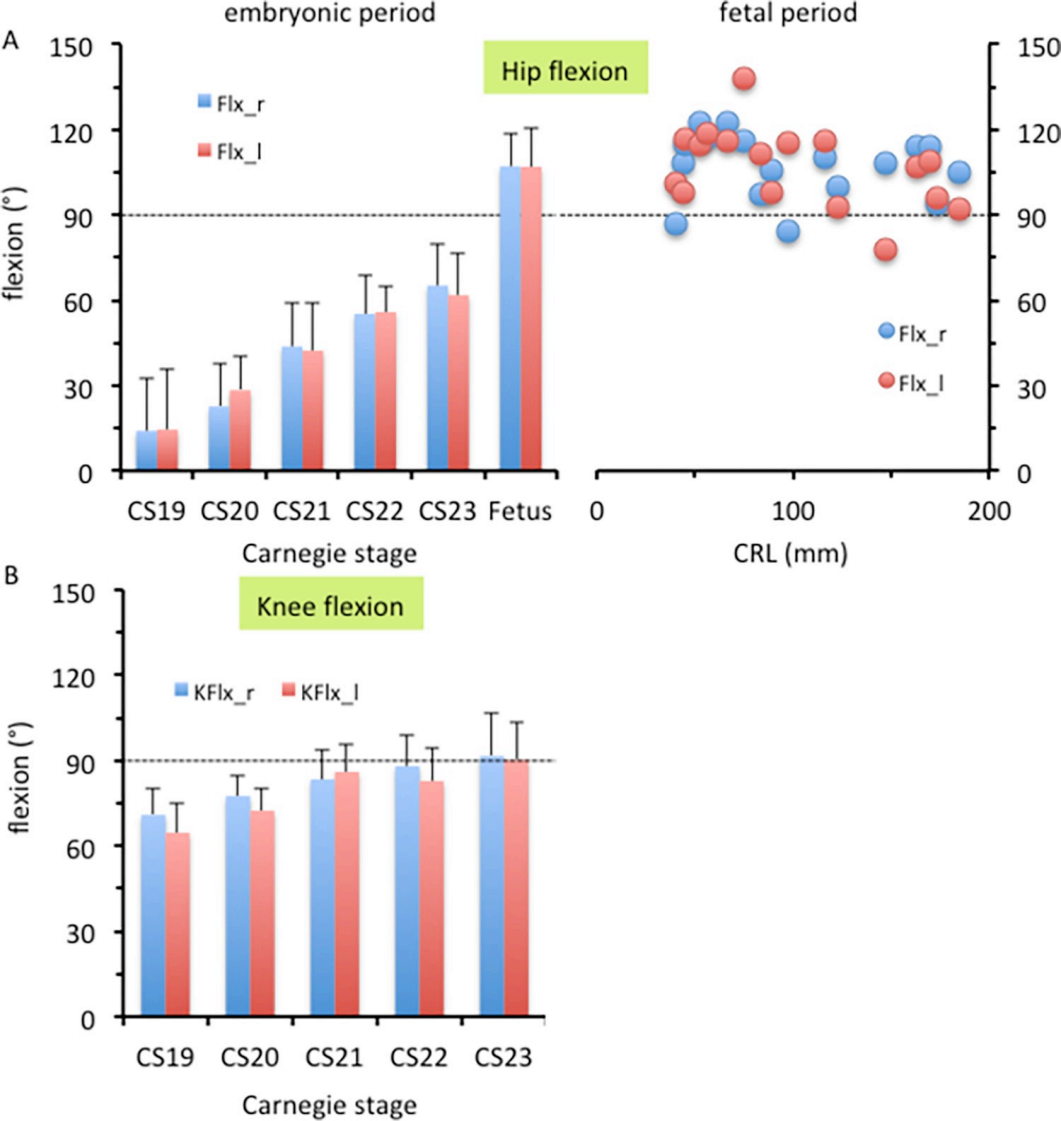
Flexion angle in the lower limbs. Hip joint (A) and knee joint (B) flexion. (A) Hip joint flexion during the embryonic (left) and fetal (right) periods. (B) Knee joint flexion during the embryonic period. Legends: blue, right lower limb; red, left lower limb.

The femur was elongated caudally, as shown in Fig 3. Hip flexion was approximately 14° at CS19 and gradually increased during the embryonic period, reaching approximately 65° at CS23 (Fig 4A). During the fetal period, the thigh was in the “sitting position,” and morphometry indicated that hip flexion increased to 107°. The hip flexion angle in most samples ranged from 90° to 120°, which was relatively constant, as compared to the hip rotation angle. Knee flexion was approximately 70° at CS19 and gradually increased to approximately 90° at CS23 (Fig 4B).

#### 3.1.2. Ventral view (abduction and lateral rotation)

**Fig 5:**
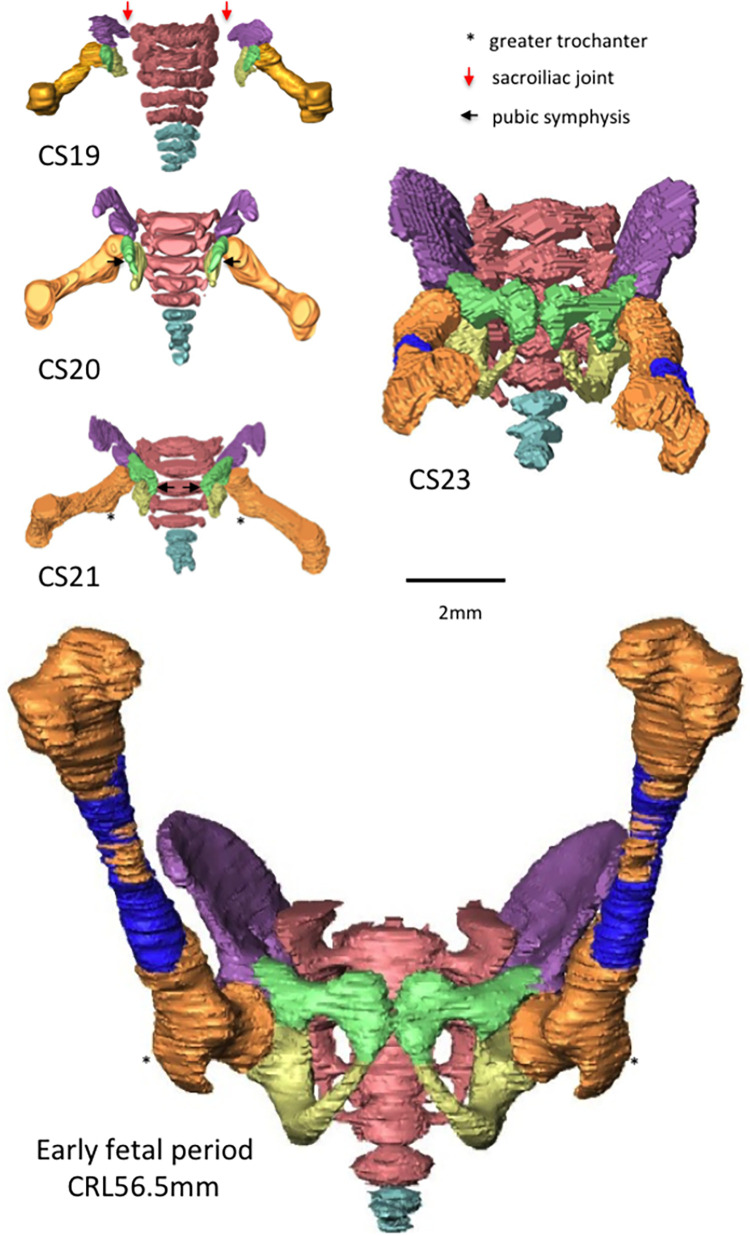
Representative 3D reconstructions of skeletal structures: The hip joint (ventral view). Note that the pubic symphysis (black arrows) was first contacted at CS23. Legends: blue, femur (ossified); brown, femur (not ossified); green, pubis; light blue, coccyx; pink, sacrum; purple, ilium; yellow, ischium. “*” indicates the greater trochanter, and the red arrows point at the sacroiliac joint.

**Fig 6:**
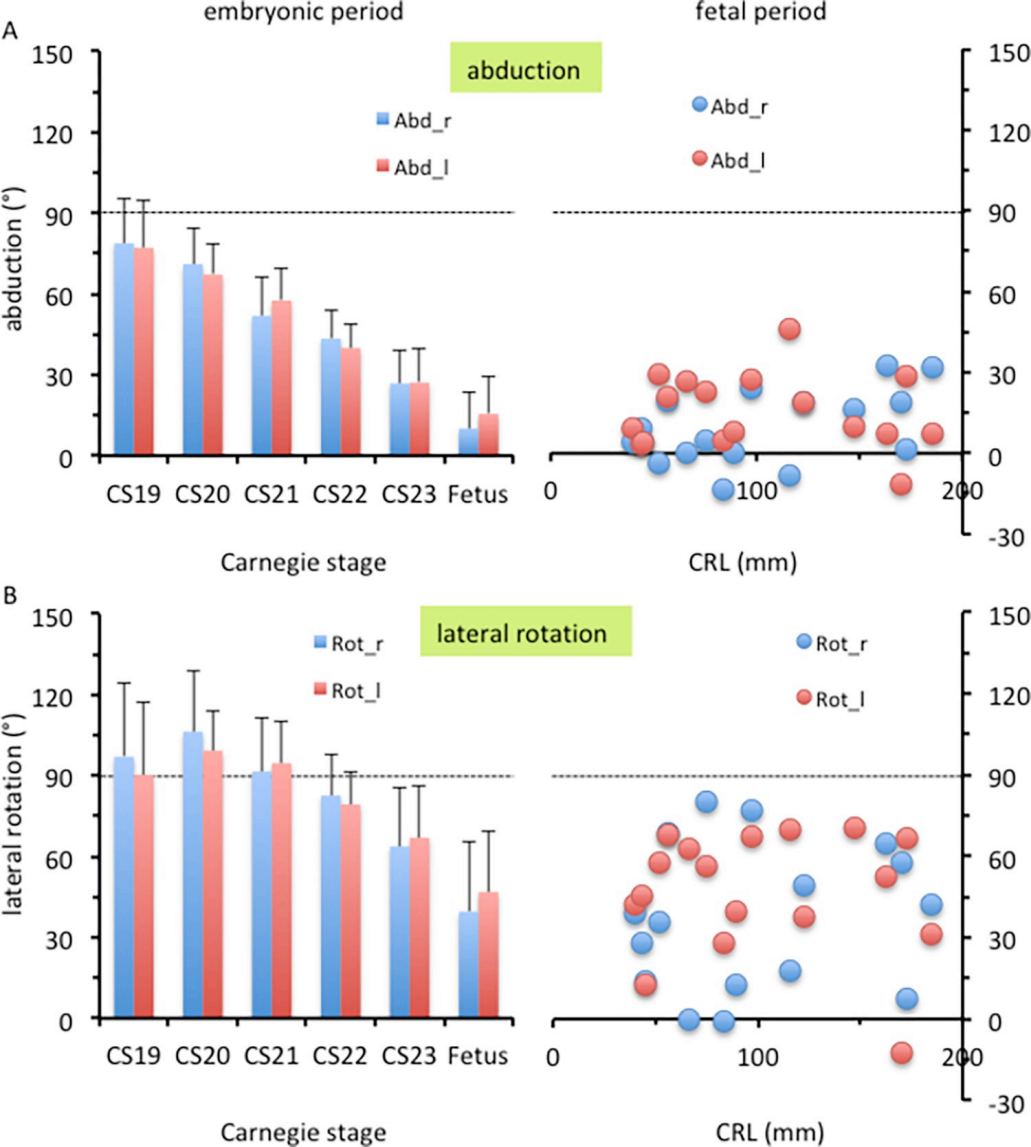
Abduction angle and lateral rotation angle of the hip. (A) Abduction angle of the hip during the embryonic (left) and fetal (right) periods. (B) Lateral rotation angle of the hip during the embryonic (left) and fetal (right) periods.

The hip joint showed abduction from the z-axis during the embryonic period (Fig 5). Abduction at the hip joint was approximately 78° at CS19 and gradually decreased to approximately 27° at CS23 (Fig 6A). The average angle during the fetal period was 10.1° on the right and 15.5° on the left. The angle ranged from 0° to 30°, which was relatively constant, as compared with the hip rotation angle. The angle measures were diverse among the samples.

At CS19 and CS21, the greater trochanter was located on the caudal side and was recognizable in the ventral view (asterisk in Fig 5), suggesting that the femur showed lateral rotation. Morphometry revealed that lateral rotation was greater than 90° at CS19 and CS21 and decreased to approximately 65° at CS23 (Fig 6B). The average angle during the fetal period was 39.6° on the right and 46.8° on the left. The angle measures were diverse among the samples.

#### 3.1.3. Comparison of three posture parameters

**Fig 7:**
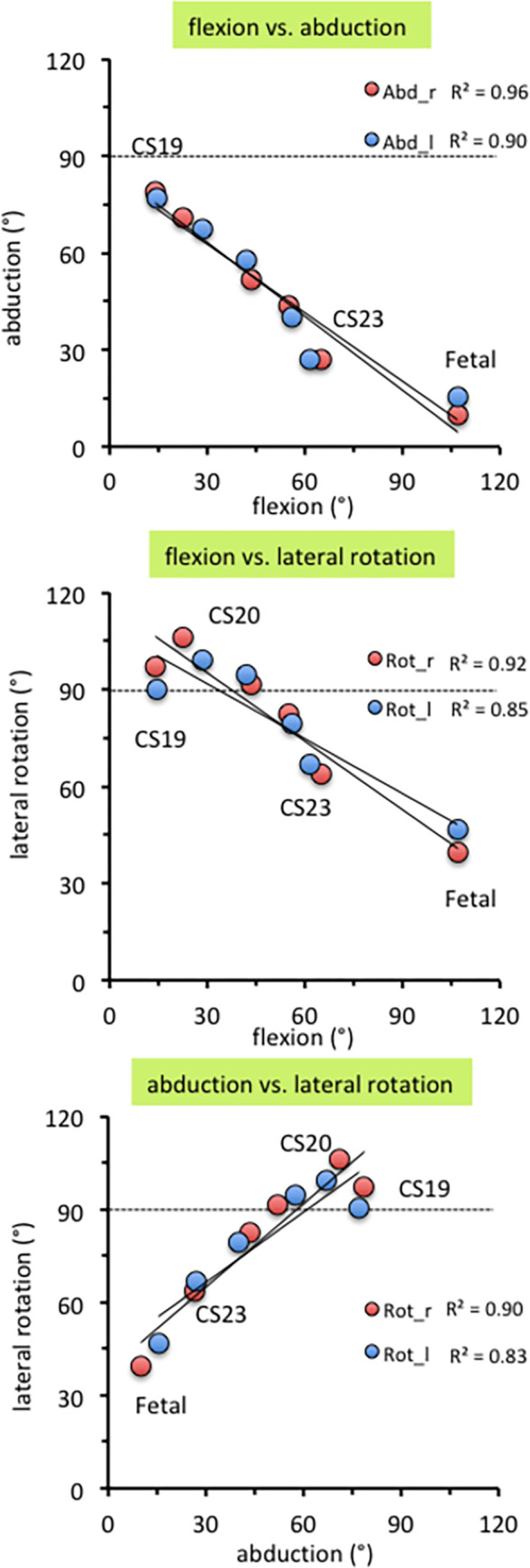
Correlation between flexion, abduction, and lateral rotation of the hip.

Three posture parameters—namely, flexion, abduction, and lateral rotation of the hip—were linearly correlated with each other at CS19 and later stages (Fig 7). The coefficient of determination (R^2^) was 0.96 and 0.90 for right and left flexion vs. abduction, 0.92 and 0.85 for right and left flexion vs. lateral rotation, and 0.90 and 0.83 for right and left abduction vs. lateral rotation, respectively.

### 3.2. Pelvic ring formation and length measurements

**Fig 8:**
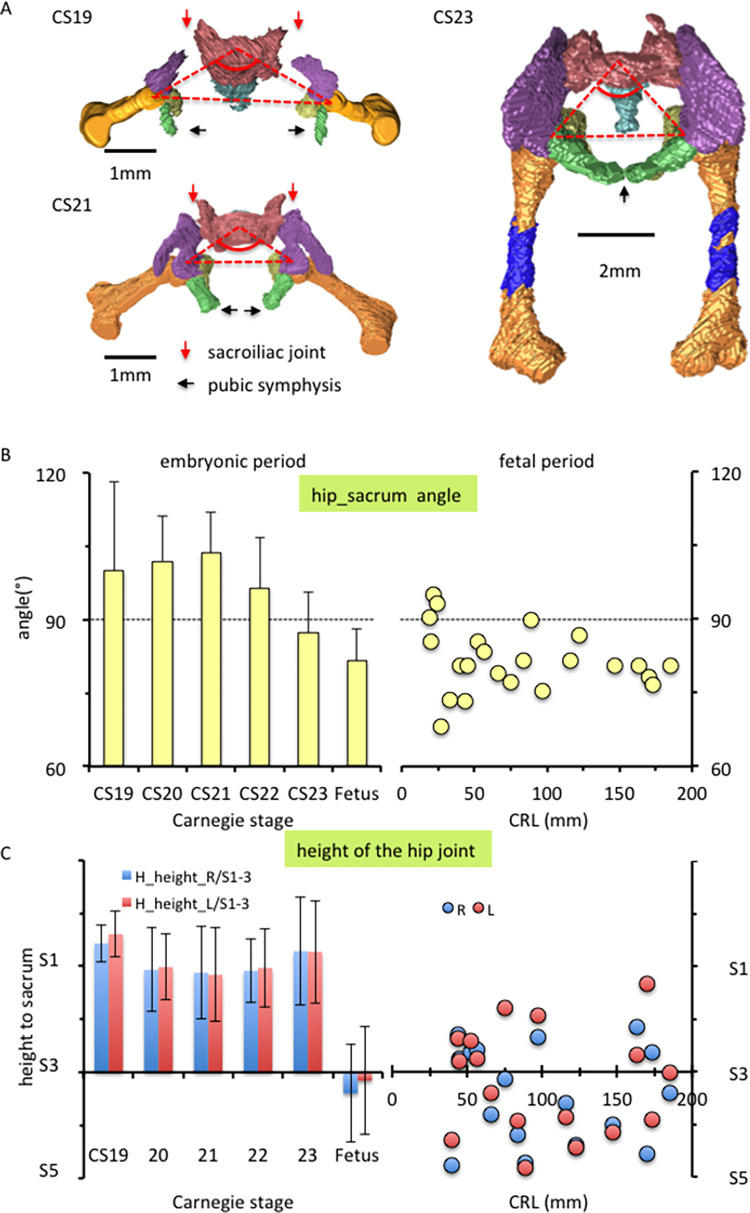
Pelvic ring formation. (A) Cranial view of the 3D reconstruction indicating pelvic ring formation during the embryonic period (CS19, CS21, and CS23). The red triangle indicates the hip–sacrum angle (i.e., the angle between the right femoral head, first sacrum, and left femoral head [∠H_r-S1 and S1-H_l]), which was projected onto the x–y plane (transverse plane). Legends: Blue, femur (ossified); brown, femur (not ossified); green, pubis; light blue, coccyx; pink, sacrum; purple, ilium; yellow, ischium. (B) Change in the hip–sacrum angle during the embryonic (left) and fetal (right) periods. (C) Height of the hip joint during the embryonic (left) and fetal (right) periods. The height of the hip joint along the z-axis (cranial–caudal direction) is shown, with the position of S3 indicated as a reference.

Prior to pelvic ring formation, the pelvis was “platypelloid.” Consequently, the hip–sacrum angle changed (Fig 8A). The angle was >100° at CS19 and CS21 but decreased to 87° at CS23 and then 82° during the fetal period (Fig 8B). This angle had a relatively narrow range in most fetal specimens (75° to 85°).

The relative position of the hip joint and sacrum in the cranial–caudal direction was noted to have changed between the embryonic and fetal periods (Fig 8C). The hip joint was located almost at the level of S1 during the embryonic period and at the level of S3 during the fetal period (see also Fig 3).

**Fig 9:**
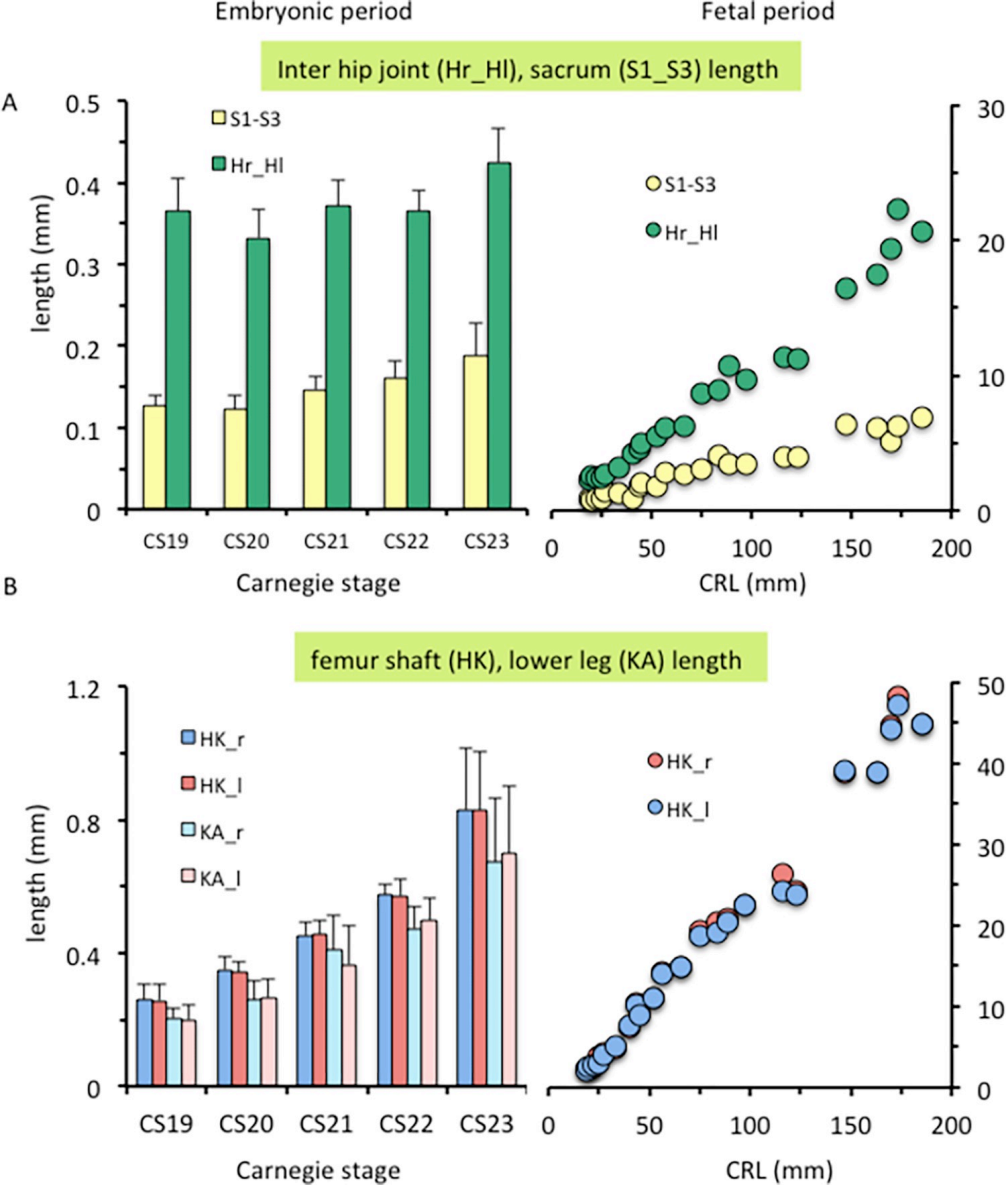
Length measurements of the lower limbs. (A) Pelvic length measurements during the embryonic (left) and fetal (right) periods: lengths of the inter-hip joint (segment H_r-H_l) and sacrum (S1–S3). (B) Length measurements of the lower limbs during the embryonic (left) and fetal (right) periods: lengths of the bilateral femoral shafts (segment H-K) and lower limbs (segment K-A).

The inter-hip joint length was almost constant (0.33–0.37 mm) from CS19 to CS22, as compared with other length measurements such as S1–S3 length, femoral shaft length, and lower-leg length (Fig 9). During the fetal period, all length measurements linearly increased according to growth.

As indicated by the ventral (Fig 5) and cranial views (Fig 8A), the pelvic ring was still open at CS19; subsequently, it was closed by sacroiliac joint formation at CS21 and pubic symphysis formation at CS23.

## 4. Discussion

The present study evaluated the femoral posture using the angle between the z-axis (body axis) and the line on the hip and knee joints. The planes of the hip, knee, and ankle joints were used to evaluate lateral rotation, as abduction and adduction of the knee joint might be limited. Our hip joint angle calculation for the evaluation of lower-limb posture is more accurate than that applied in a previous study [3], in which toe and foot orientations were used as reference points.

Changes in lateral rotation, adduction, and flexion during the embryonic period were -37.4°, -51°, and 49.2°, respectively. Our results were inconsistent with the description in several textbooks that 90° rotation occurs at eight weeks of gestation [16–19]. O’Rahilly and Gardner [3] reported that the pre-axial border remained cranial at the end of the embryonic period proper (CS23), on the basis that the big toe was located on the cranial side and the foot sole was oriented medially at CS23. It should be noted that the lower-limb structure of an embryo or fetus is different from that of an adult. For instance, the “physiological clubfoot” is recognized during the embryonic and early fetal periods (Fig 1) [8, 9]; therefore, the foot’s orientation should not be used as an indicator of lower-limb posture.

Three posture parameters—namely, flexion, abduction, and lateral rotation of the hip—were linearly correlated with each other during the embryonic period, suggesting that the femoral posture at each stage was three-dimensionally constant and gradually and smoothly changed according to growth. During the fetal period, these posture parameters varied among individuals, especially the lateral hip rotation angle. No obvious trend in this variation was observed. When the joint cavity develops and a pelvic ring structure is formed during the fetal period, the hip joint may be actively movable in the uterus. Thus, the posture of the preserved fetal samples might not accurately reflect the intrauterine posture.

We recently presented data on upper-arm posture [15], as well as the morphogenesis and position of the scapula [20]. There were similarities in posture between the upper arms and thighs, as described in the present study. A gradual change in posture was observed during the embryonic period, in which the maximum value was attained at CS19 or CS20; in contrast, the posture was relatively constant during the fetal period. For both upper and lower limbs, individual differences were large during the fetal period. With respect to the abovementioned three parameters, a decrease in abduction was observed in both upper and lower limbs. Unfortunately, this gradual change in posture may be unsuitable as a parameter for staging, which requires a specific form in appearance.

On the other hand, the following differences in posture were observed between the upper and lower limbs. Decreased lateral rotation was evident in the lower limbs but was inconspicuous in the upper limbs. As for flexion, the angle in the lower limbs increased during the embryonic period and was constantly high during the fetal period, whereas the angle in the upper limbs was relatively constant (45° to 90°). It should be noted that the position of the scapula influences the upper-arm posture. The position of the scapula considerably changed and was significantly different between the embryonic and fetal periods. Abduction and flexion of the scapulothoracic articulation were significantly increased, which affected the upper-limb posture. Regarding the lower limbs, the sacropelvic joint was formed at CS21 and the pubic symphysis was observed at CS23 (Figs 5 and 8A). Pelvic ring formation may influence the abduction angle in the lower limbs; nevertheless, such influence may be less than the influence of the scapula on the upper-limb posture. However, in the present study, we could not clarify the effect of pelvic ring formation on the lower-limb posture.

The hip joint consists of the femoral head and pelvis. The pelvis and femur are connected, and the border can be recognized in samples through the presence of dense mesenchymal cells at CS21 and earlier stages. Although they are still connected, the density of mesenchymal cells decreases between CS22 and CS23. The Y-shaped connection of the three parts of the hip bone (namely, the ischium, pubis, and ilium) forms the acetabulum at approximately CS23 [6, 21, 22]. A previous study reported that cavitation was initiated in samples with a CRL of ≥30 mm and was always observed in samples with a CRL of ≥56 mm [6]. Changes in the hip joint posture during the embryonic period may cause considerable stress on the musculoskeletal system of the hip joint, considering that the pelvis and femur are connected. Future studies should investigate the effects of stress on this system.

The trochanter initially forms at approximately CS21, and the femoral neck–shaft angle is observed at the end of the embryonic period. The angle ranges from approximately 135° to 145° before femoral ossification (from CS23 to the early fetal period) and is approximately 130° during the fetal period, which is similar to that in adults [7]. Furthermore, the detected anteversion angle is approximately 25° before ossification and ranges from 10° to 20° during the fetal period. These angles can potentially influence each other in a complex manner. Ideally, the angle should be measured at the femoral shaft and neck separately. However, in the present study, setting the proper landmarks on the trochanter to accurately separate the femoral neck and shaft was difficult to accomplish because of the low MRI resolution and trochanter’s immature form during the embryonic period.

Using 3D ultrasound in virtual reality, Bogers et al. recently analyzed the physiological development of the fetal foot position “physiological clubfoot” during the first trimester [9]. Their study pointed out several issues for the applicability of their angle measurement technique to an embryonic study in the first trimester. The skeletal system during the embryonic period mainly comprises cartilages, which are less echogenic, and the lengths of the cartilaginous fibula and tibia could not be measured in their study. Limb posture measurements depend on the surface view, which may reduce the accuracy. The success rate of angle measurements are still low (13.5–25.7%) for embryos at 8–9 weeks of gestational age. We consider that the hip joint will be much more difficult to accurately estimate using *in vivo* ultrasound images. The proportion of the trunk and thighs, which are not so long in the cranial–caudal direction, will not be suitable for accurate measurements of the hip joint length and angle if the skeletal system is not detected. In our opinion, accurate limb measurements using ultrasound images may be applicable during the fetal period when the body size becomes large, the limbs elongate longitudinally, and the bone structure begins to be detected.

The use of high-resolution MRI provides images of early anatomical development to improve the understanding of lower leg posture during the embryonic and early fetal period. This study also evaluates imaging data using the Carnegie collection [23] and expands on results from previous studies [1, 3]. Dawood et al. [24] highlighted the importance of ex utero imaging techniques, such as micro-CT and high-resolution MRI, to facilitate proper annotation of fetal structures in ultrasound scans of early first-trimester embryos and to improve detection of congenital anomalies in the developing embryo. From this point of view, our present study may help address these concerns and contribute to the improvement of the prenatal medicine.

### 4.1. Limitations

This study has some limitations. First, deformities and shrinkage due to fixation and preservation should be considered, as these may affect the limb posture, particularly in fetal samples after the formation of hip joint cavitation and pelvic ring. Second, although our samples were classified as normal based on external morphology, we could not unequivocally ascertained that all samples underwent normal development. Finally, staging between CS19 and CS23 was considerably difficult; however, we carefully staged the embryonic period samples, as previously described.

### 4.2. Conclusions

The angle calculation in our present study is more accurate than previous measures; hence, evaluating the femoral posture through our method is more reliable, revealing the precise timeline of femoral posture changes. The femoral posture at each embryonic stage was three-dimensionally constant and changed gradually and smoothly according to growth. The findings of our study may be difficult to apply clinically, however, our study has merits in that lengths and angles were measured on anatomical landmarks of the skeletal system. Our obtained data may contribute to an understanding about development from anatomical aspects and provide valuable insights for clinical application.

## Supporting information

S1 Datasets Length and angle values. (XLSX)
